# Investigating the potential of dietary iron supplementation to enhance long-chain polyunsaturated fatty acid biosynthesis in *Hediste diversicolor*

**DOI:** 10.1038/s41598-025-26488-x

**Published:** 2025-11-27

**Authors:** Andrea Villena-Rodríguez, Juan C. Navarro, Inmaculada Varó, Felipe Aguado-Giménez, Júlia Pérez-Ara, Myriam Lizanda, Ignacio E. Martín, Inmaculada Rasines, Óscar Monroig

**Affiliations:** 1 Instituto de Acuicultura Torre de la Sal (IATS), CSIC, Ribera de Cabanes, Castellón, 12595 Spain; 2 Planta de Cultivos Marinos “El Bocal”, Centro Oceanográfico de Santander. Instituto Español de Oceanografía (COST‑IEO), CSIC, Monte‑Corbanera, Santander, 39012 Spain

**Keywords:** Desaturases, Fe supplementation, *Hediste diversicolor*, LC-PUFA biosynthesis, Biochemistry, Physiology

## Abstract

Fatty acyl desaturases are nutritionally regulated and depend on iron (Fe) for optimal activity. We hypothesised that dietary Fe supplementation, under conditions promoting desaturase upregulation, can enhance desaturase activity and improve n-3 LC-PUFA biosynthesis in invertebrates. The present study aimed to evaluate the effects of dietary Fe supplementation on LC-PUFA biosynthesis in *H. diversicolor*, a commercially important polychaete, using both *In vitro* and *in vivo* approaches. The *in vitro* assays revealed that Fe supplementation, from both inorganic (FeSO_4_) and organic (ProPath^®^ Fe) sources, has potential to increase desaturase activity, particularly on the *H. diversicolor* methyl-end desaturase ωx2, under high-expression conditions simulated in yeast. In contrast, *in vivo* feeding trial showed no significant differences in LC-PUFA composition of *H. diversicolor* fed Fe-supplemented diets compared to controls. This apparent discrepancy may be due to the LC-PUFA-free diet not sufficiently upregulating fatty acyl desaturases, leading to no increased Fe demand for enzyme activity. Alternatively, although the LC-PUFA-free diet may have triggered desaturase upregulation, the expected effects of Fe supplementation on desaturase activity and LC-PUFA content were not observed. This suggests Fe was not a limiting factor, even in the control group.

**Supplementary Information:**

The online version contains supplementary material available at 10.1038/s41598-025-26488-x.

## Introduction

In recent years, alternative and sustainable ingredients for aquafeed have been extensively evaluated to reduce the dependence on fishmeal (FM) and fish oil (FO)^1–3^. Among them, ingredients derived from marine invertebrate biomass, particularly polychaete worms, have emerged as promising candidates due to their high contents of essential nutrients like long-chain (≥ C_20_) polyunsaturated fatty acids (LC-PUFAs), including the n-3 (omega-3) eicosapentaenoic acid (EPA, 20:5n-3) and docosahexaenoic acid (DHA, 22:6n-3), and the n-6 (omega-6) arachidonic acid (ARA, 20:4n-6)^4–7^. Interestingly, their nutritional value with high LC-PUFA levels, combined with detritivorous feeding habits, enable polychaetes to be cultivated on waste materials (or “sidestreams”) from various bioindustries. Several studies have demonstrated that the nereid polychaete *Hediste diversicolor* can feed on a wide range of side streams, including sludge from land-based aquaculture and the solid residue left after biogas production, known as “solid biogas digestate”^4,6,7^. Although these sidestreams are typically low in LC-PUFAs, *H. diversicolor* grown on them exhibit relatively high LC-PUFA contents^4,7^. These observations suggest the ability of *H. diversicolor* for LC-PUFA biosynthesis, a hypothesis confirmed by other studies that demonstrated this species possesses the complete enzymatic machinery required for LC-PUFA biosynthesis^8,9^. The enzymatic complement enabling animals to biosynthesise LC-PUFAs, on one hand, elongation of very long-chain polyunsaturated fatty acid (Elovl) proteins, responsible for the extension of the fatty acid (FA) substrate chain, and, on the other, fatty acyl desaturases, which introduce new double bonds (unsaturations) into the FA substrate chain. Notably, *H. diversicolor* has unique desaturase enzymes termed “methyl-end” (or “ω”) desaturases, which enable *de novo* biosynthesis of PUFA and, from them, LC-PUFAs^8^. Specifically, the enzyme referred to as “ωx1” was found to exhibit both ω6 and ω3 desaturation activities. Additionally, another methyl-end desaturase, termed “ωx2” was identified as an ω3 desaturase, an enzyme that converts various n-6 PUFA substrates into their corresponding n-3 PUFA products^8^. Along with methyl-end desaturases, *H. diversicolor* has other fatty acyl desaturases known as “front-end desaturases” (Fed)^9^; in particular, the so-called “Fed1” is a Δ5 desaturase, whereas the *H. diversicolor* “Fed2” has Δ6 and Δ8 desaturase activities^9^.

Fatty acyl desaturases including methyl-end and front-end desaturases involved in animals’ LC-PUFA biosynthesis have demonstrated to be functionally diverse^10^. Their activity can be modulated at different levels via diet (nutritional regulation) and environmental factors such as temperature and salinity^11^. Importantly, the efficiency of fatty acyl desaturases is dependent on the availability of trace elements, among which iron (Fe) plays particularly relevant roles^12^. Fe acts as a co-factor in the active di-iron centres of fatty acyl desaturases and serves as a key structural component of the desaturase enzyme complex^13,14^. Previous studies in humans have demonstrated that Fe deficiency has an adverse effect on PUFA metabolism, since it affects their synthesis through Δ6 desaturase activity^15^. Similarly, several studies in fish including the rainbow trout (*Oncorhyncus mykiss*)^16,17^ and Atlantic salmon (*Salmo salar*)^18^, have reported that dietary Fe supplementation may have a positive effect on LC-PUFA biosynthesis, increasing the n-3 LC-PUFA contents and modulating the activity of the fatty acyl desaturase Fads2. However, to the best of our knowledge, the effects of Fe supplementation as a strategy to boost the LC-PUFA biosynthesis in aquatic invertebrates has not yet been investigated.

The bioavailability, absorption and utilisation of trace minerals are influenced by their chemical form. Traditionally, trace elements have been added to animal feeds as inorganic salts. However, due to the potential limitations in mineral availability from these sources, alternative forms including organic or chelated trace minerals, have been explored^19,20^. Numerous studies in fish have shown increased bioavailability of trace minerals from chelated sources compared to inorganic salts^21–24^. Studies investigating the beneficial effects of organic versus inorganic minerals in invertebrates are scarce, with most focusing on commercially important crustaceans such as *Litopenaeus vannamei*^25–28^ and the mud crab *Scylla paramamosain*^29^. However, research on other groups, such as annelids, is very limited, especially when referring to Fe.

Based on the capacity of invertebrates’ fatty acyl desaturases to be nutritionally regulated^30^ and their dependence on Fe for optimal activity, we hypothesise that dietary Fe supplementation to invertebrates cultured under conditions that promote the up-regulation of fatty acyl desaturases will enhance desaturase activity. This would occur by preventing a limitation of enzyme co-factor (Fe), whose demand is expected to increase due to the higher number of desaturase molecules produced as a result of the regulatory factors triggering the up-regulation. Enhancement of desaturase activity, in turn, can improve the n-3 LC-PUFA biosynthetic capacity of invertebrates and enhance the nutritional profile of their biomass for potential use as feed ingredients. Therefore, the present study aimed to evaluate the effects of dietary Fe supplementation as a potential booster of LC-PUFA biosynthesis in the nereid polychaete *H. diversicolor*, a commercially important species with significant aquaculture potential. Using both *in vitro* and *in vivo* approaches, we investigated the influence of Fe supplied as an inorganic salt (FeSO_4_) or as an organic Fe source (ProPath^®^ Fe), on LC-PUFA biosynthesis and the expression of *H. diversicolor* fatty acyl desaturase enzymes^8,9^.

## Materials and methods

### *In vitro* assays to test the efficiency of Fe on desaturase activity

The *in vitro* assays aiming to determine the efficiency of Fe supplementation on desaturation activity were conducted using a heterologous expression system consisting of yeast (*Saccharomyces cerevisiae*) expressing one of the four desaturases previously reported in *H. diversicolor*^8,9^. The plasmid constructs designed for functional characterisation of these desaturases, included pYES2-Fed1 and pYES2-Fed2 for the two front-end desaturases^9^, and pYES2-ωx1 and pYES2-ωx2 for the two methyl-end desaturases^8^. These plasmids were transformed into competent *S. cerevisiae* INvSc1 cells (Thermo Fisher Scientific) using the S.C. EasyComp^®^ Transformation kit (Thermo Fisher Scientific). The transformed yeast were selected on *S. cerevisiae* minimal medium minus uracil (SCMM^−URA^) agar plates and incubated at 30 °C for 3 d. A single recombinant colony from each gene transformation was cultured in SCMM^−URA^ broth at 30 °C for 2 d to achieve an OD600 between 8 and 10, following the procedure described by^31^. Then, the suitable volume of the yeast cultures was inoculated in 5 mL of SCMM^−URA^ broth within individual 150 mL Erlenmeyer flasks to provide an OD600 of 0.4. The recombinant yeast in each flask was grown at 30 °C with constant shaking (250 rpm) for 4 h until reaching an OD600 of approximately 1. At that point, the transgene expression was induced by supplementing the culture with galactose at 2% (w/v), and one of the potential FA substrates. The exogenously supplied FA substrates varied among the assayed desaturases, according to the substrate affinity shown in previous studies^8,9^.

The overall protocol was conducted following procedures similar to those described in^8,9^. For the *H. diversicolor* Fed, the selected FA substrates were 20:3n-6 (Δ5 desaturase substrate) for Fed1 and 20:2n-6 (Δ8 desaturase substrate) for Fed2. Moreover, the selected FA substrate for the *H. diversicolor* methyl-end desaturases was 18:2n-6 since this FA is an adequate substrate to test the Δ15 desaturation activity exhibited by both *H. diversicolor* ωx1 and ωx2^8^. For each of the four *H. diversicolor* desaturases, the following four treatments were assayed: no Fe supplementation (control), supplementation with either FeSO_4_ (Inorganic Fe) or ProPath^®^ Fe (Organic Fe), and supplementation with an Fe chelating agent (bathophenanthroline disulfonate, BPS) (chelator). Four replicates per treatment were assayed. As Inorganic Fe, FeSO_4_.7H_2_O (iron (II) sulfate heptahydrate, ≥ 99%, Sigma-Aldrich) was used. The organic Fe consisted of the ProPath^®^ Fe kindly provided by Zinpro (Zinpro Corp., USA) declared as iron chelate of lysine and glutamic acid (iron amino acid complex) according to Commission Regulation (EU) 2020/1795. This product contains 15% iron (w/w) and approximately 18–22% lysine and 18–22% glutamic acid, with a crude protein content of 36% and ash content of 31%. The compound is a stable 1:1 complex in which each iron ion is bound to the amino acids lysine and glutamic acid, resulting in a highly bioavailable organic iron source. Stock solutions of 100 mM Fe of both inorganic and organic Fe sources were prepared in 0.1 N HCl, while that of the Fe chelating agent BPS was prepared in distilled water at 100 mM. All substrates were supplemented as sodium (Na) salts at final concentrations of 0.5 mM (C_18_) and 0.75 mM (C_20_) as uptake efficiency decreases with increasing chain length FA substrates (> 98–99% pure). All FAs were obtained from Nu-Chek Prep, Inc. (Elysian, MN, USA). Yeast culture reagents including galactose, nitrogen base, raffinose, tergitol NP-40, stearidonic acid (> 99%) and uracil dropout medium were obtained from Sigma-Aldrich. The yeast cultures (comprising 4 150-ml flasks per treatment x 4 treatments) were incubated at 30 °C and continuous shaking at 250 rpm for 2 d. Following incubation, the yeast cells were collected by centrifugation at 2000 g for 2 min. The resulting pellets were washed twice with 2.5 mL of double-distilled water (ddH_2_O), then homogenised in 1 mL of 2:1 (v/v) chloroform: methanol containing 0.01% (w/v) butylated hydroxytoluene (BHT, Sigma-Aldrich) as antioxidant. An additional 5 mL of 2:1 (v/v) chloroform: methanol was added, and the samples were stored at −20 °C under oxygen-free conditions for at least 24 h until further analysis, according to the method described by^9^.

### *In vivo* feeding trial to evaluate the effect of Fe on desaturase activity

**Fig. 1:**
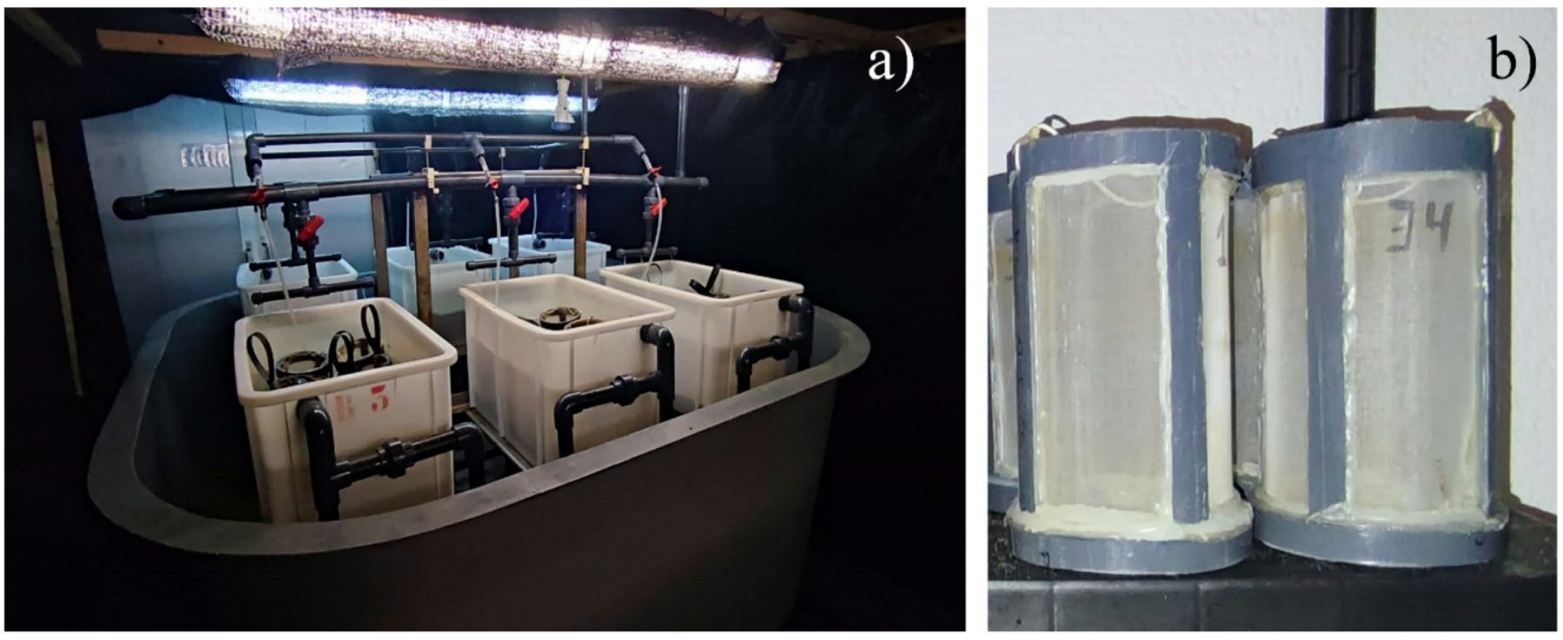
**(a)** RAS, including three polycarbonate trays (width 35 cm; height 30 cm; length 54 cm); **(b)** Experimental units (height 20 cm; internal diameter 11.3 cm).

The following experimental setup was conducted using a protocol similar to that described in previous studies^32,33^. Polychaetes (*H. diversicolor*) of 25–50 mg wet weight from a captivity inbred stock held at the Oceanographic Centre of Santander (COST‑IEO-CSIC) were randomly distributed in groups of three experimental units (3 units/diet) with 20 worms each (biological replicates). Prior to the experiment, the worms had been fed on fish feed for *Solea senegalensis* (0.35–0.50 mm pellets; Skretting GEMA NEO). Each experimental unit consisted of cylindrical PVC frame (20 cm in height; 11.3 cm internal diameter) with walls and bases made of 335‑µm mesh (Fig. 1). These frames were filled up with sieved sand (grain size 0.25–1 mm) up to a height of 12 cm, as previously validated in^32,33^. These experimental units were placed inside polycarbonate trays (35 cm width, 30 cm height, and 54 cm length, with a usable volume of 51 L), that formed part of a recirculating aquaculture system (RAS). This system included mechanical and biological filtration, UV sterilisation and temperature regulation via an Eliwell eWPC‑800T electric heater and a HAILEA HC500‑A water cooler (Fig. 1). Each tray was maintained with a recirculating water flow of 1.5 L min^− 1^, with approximately 10% of the total water volume in the RAS renewed daily. The experimental units were partially submerged in the trays, allowing the sediment layers to remain underwater while the upper parts of the units extended above the water level. To ensure effective water exchange and prevent the loss of both worms and feed, each tray was equipped with an overflow mechanism below the upper level of the experimental units. Environmental conditions were controlled, maintaining a salinity level set to 36‰ and a photoperiod of 16 L: 8D, as described in^32,33^.

The worms were fed on the corresponding experimental diet lacking LC-PUFAs for 49 d. The Inorganic Fe (iron (II) sulfate heptahydrate) contained 20% Fe, whereas the Organic Fe (ProPath^®^ Fe) contained 15%. Both Fe-supplemented diets were formulated with 675 mg kg^− 1^ of their respective Fe source, resulting in calculated Fe concentrations of approximately 135 mg Fe kg⁻^1^ diet (Inorganic Fe) and 101 mg Fe kg⁻^1^ diet (Organic Fe). The slightly lower inclusion level in the Organic Fe treatment accounts for the higher bioavailability of the organic source, as chelated forms of Fe are generally more stable in the digestive tract and more efficiently absorbed and utilized by organisms^19–24^. The Control diet contained no supplemental Fe. These calculated Fe concentrations have been included in Suppl. Table S1. Details of the ingredient composition and FA profiles of the experimental diets are shown in Suppl. Tables S1 and S2. The experimental diets were manufactured by LifeBioencapsulation S.L. (Almería, Spain). Worms were fed to 4% of the biomass 5 d per week. At the end of the experiment, polychaetes were dug out of the experimental units and starved for 24 h prior sampling. Survival and growth performance were estimated. Specific growth rate (SGR) was calculated using the average wet weight of worms in each treatment group at the beginning and the wet weight of the remaining worms at the end of the experiment. For SGR calculation, we used the formula SGR = (ln wet weight t_end_ – ln wet weight t_0_)/t, where t_0_ and t_end_ are the weight of the polychaetes at the beginning and at the end of the experiment, respectively, and t is the time at the end of the experiment (49 d). From each diet (3 experimental unit replicates), 60 polychaetes were randomly sampled. A total of 30 individuals (10 per replicate experimental unit) were frozen at −80 °C until they were freeze-dried before shipment from COST‑IEO-CSIC to the facilities of IATS-CSIC (Ribera de Cabanes, Spain) for FA analysis. Similarly, 30 further polychaetes (10 per replicate experimental unit) were sampled as described above and immediately preserved in RNAlater (Thermo Fisher Scientific, Waltham, MA, USA) for gene expression analyses.

### Fatty acid analysis

Total lipids (TL) were extracted from the homogenised yeast samples using a modified Folch method^34^. Briefly, yeast pellets were suspended in chloroform-methanol (2:1, v/v) containing 0.01% BHT as antioxidant, and thoroughly homogenized. Phase separation was induced by adding 0.88% KCl solution, followed by centrifugation. The lower organic phase was carefully collected, washed once with chloroform-methanol (2:1, v/v), and subsequently evaporated to dryness under a gentle stream of nitrogen using an analytical evaporator. The extracted lipids were weighed and stored at −20 °C until further analyses. Fatty acid methyl esters (FAMEs) were prepared from yeast TL extracts through acid catalysed transmethylation, and purified using thin layer chromatography on 20 × 20 cm plates (Merck KGaA, Darmstadt, Germany)^35^. For the *H. diversicolor* samples derived from the *in vivo* trial, six individuals (*n* = 6; two per each replicate experimental unit) per dietary treatment (Control, Inorganic Fe and Organic Fe) were analysed. Extractions of TL from pre-weighed freeze-dried whole-body samples were carried out following the same methodology as described above for yeast, and an aliquot of TLs (500 µg) was used to prepare FAMEs as described above^35^. FAMEs from either yeast (*in vitro* assays) or *H. diversicolor* (*in vivo* feeding trial) were analysed using a Thermo Trace GC Ultra Gas Chromatograph (GC) (Thermo Electron Corporation, Waltham, MA, USA). The instrument was equipped with an on-column injection system, and a flame ionisation detector (FID), using helium as the carrier gas. Separation was carried out using a 30 m×0.25 mm fused silica open tubular column (Tracer, TR-WAX, film thickness: 0.25 μm, Teknokroma, Sant-Cugat del Vallés, Spain). The GC oven temperature was programmed to rise from 50° C to 220° C. Chromatograms were integrated and analysed using Azur Datlys software (St Martin d’Heres, France). Fatty acid standards were purchased from Supelco (37 Component FAME Mix, 10 mg/mL, Sigma-Aldrich, Bellefonte, PA, USA). In addition, FAMEs were identified by comparison with a well-characterised sardine oil standard (Marinol^®^, DSM Nutritional Products, Switzerland). For further confirmation of peak identities, selected FAMEs were analysed using an Agilent 6850 Gas Chromatograph system coupled to a 5975 series MSD (Agilent Technologies, Santa Clara, CA, USA) (GC-MS)^8,9,31^. For the *in vitro* assays, conversions of each PUFA substrate to the corresponding desaturation product were calculated by the proportion of substrate converted to a product as [area of product/(area of product + substrate area)] × 100. The FA contents of the *H. diversicolor* from the feeding trial were expressed as percentages of total FAs. All analytical procedures followed methodologies previously reported in^8,9,36^.

### Gene expression

Total RNA extraction, cDNA synthesis, and qPCR analyses were performed following the optimized protocols for *H. diversicolor* described by^36^. Briefly, total RNA was isolated from the head of single individuals (*n* = 12 samples per treatment), using RNeasy Plus Universal Mini Kit (Qiagen) following the manufacturer’s recommendations. RNA quality and quantity were checked by 1% (w/v) agarose gel electrophoresis and spectrophotometry (Nanodrop ND-2000 C, Thermo Fisher Scientific, Barcelona, Spain). Samples were collected from the three independent experimental units per treatment (20 worms per unit). For complementary DNA (cDNA) synthesis, two heads from different individuals belonging to the same treatment were pooled, resulting in six pooled samples (*n* = 6) per treatment. Each pool therefore represented RNA obtained from two worms, and these pools were used as analytical replicates for qPCR analysis. cDNA was synthesised from each pooled sample, containing 1 µg of total RNA, using random primers and Moloney Murine Leukemia Virus Reverse Transcriptase (M-MLV RT, Promega). Gene expression was determined by real-time quantitative PCR (qPCR) using a qPCR thermocycler (CFX Connect Real-Time System, Bio-Rad Laboratories S.A., Madrid, Spain). The expression levels of the two front-end desaturases (*fed1* and *fed2*) and the two methyl-end desaturases (*ωx1* and *ωx2*) from *H. diversicolor* were determined using specific primers listed in Table 1. Additionally, three candidate housekeeping genes termed as β-actin (*actb*), glyceraldehyde 3-phosphate dehydrogenase (*gapdh*) and 18 S ribosomal RNA (* 18 s rRNA*), were evaluated as potential reference genes (Table 1). Primers design was carried out using Primer3 software (version 4.1.0; https://primer3.ut.ee/). The qPCR cycling program consisted of an initial activation step at 95 °C for 15 min, followed by 40 amplification cycles: denaturation at 95 °C for 15 s, annealing at 60 °C for 45 s, elongation at 72 °C for 20 s, and a final melting curve of 0.5 °C increments from 60 °C to 95 °C, confirming the specificity of amplification products. Each cDNA sample was run in duplicate, using a 1/20 dilution for target genes, and 1:30 dilution for reference genes. Reactions were carried out in a final volume of 20 µl, containing 2 µl of diluted cDNA, 0.4 µl of each primer (10 µM), 4 µl Master Mix qPCR No-ROX PyroTaq EvaGreen 5x (Cultek, Madrid, Spain), and 13.2 µl of nuclease-free ddH_2_O. Standard curves for quantification were generated on each plate using serial dilutions of pooled cDNA (ranging from 1:5 to 1:3125), run in duplicate. Negative controls without template were included on each plate to confirm absence of contamination. Amplifications efficiencies ranged between 90% and 110%. Based on NormFinder results, *actb* and *gapdh* were selected as the most stable reference genes and used for normalising the expression of target genes. Gene expression data are presented as mean normalised values ± standard deviation (SD) representing the ratio of transcript copy numbers of the *H diversicolor* fatty acyl desaturase genes (*fed1*, *fed2*, *ωx1* and *ωx2*) relative to the geometric mean of the reference genes (*actb* and *gapdh)*. All qPCR analyses were performed following the conditions optimised for *H. diversicolor* described in^36^.

**Table 1:** Primers used for real-time quantitative PCR (qPCR). Sequences of the primer pairs used (Forward: F; reverse: R), annealing temperatures (Ta), size of the fragments produced, and accession number of the sequences used for the primer design are shown.

Transcript	Primer	Primer sequence (5’−3’)	Ta	Fragment size		Accession No.
*fed1*	F	AGGATTGCTCCTCTGGTCAA	60 °C	165 bp	OQ941903	
	R	TTTGTTTCAATGCGAATCCA				
*fed2*	F	ATTTTGATGCCGGTGATGAT	60 °C	182 bp	OQ941904	
	R	CTCGCAGACAGACTTGACCA				
*ωx1*	F	TCCGCTGTTCATCTTCTCCT	60 °C	198 bp	MH469733.1	
	R	ATGTGGGTGAACATGTGGTG				
*ωx2*	F	CGACCAATGGGATATGGTTC	60 °C	165 bp	MH469734.1	
	R	TACTTCTGGCGGAAAACTGC				
*actb*	F	AGGATCTGTACGCCAACACC	60 °C	191 bp	GKPK01000000	
	R	CTGCTGGAAGGTGGAGAGAG				
*gapdh*	F	GGATCTGTTGGAGCCGATTA	60 °C	174 bp	KX284894.1	
	R	GGTCATGGATGGCTCGTACT				
* 18 s rRNA*	F	GAGTCGTAGGTGGCAAGGTG	60 °C	166 bp	GKPK01000000	
	R	TTGCACATGCATGGCTTAAT				

### Data analysis

One-way ANOVA (Tukey’s multiple comparisons, *p* ≤ 0.05) was used to analyse SGR and survival. One-way ANOVA (Dunnett’s multiple comparisons test, *p* < 0.05) was performed for the FA analysis in the *in vitro* trial, and for the gene expression by qPCR using the statistical package SPSS v.29. For the ANOVA, Diet” (Control, Inorganic Fe and Organic Fe) was the factor. Multivariate analyses of the FA profiles were carried out using both PAST (vers. 4.09)^37^ and OriginPro v.2023. Prior to the analyses, data were tested for normality (Shapiro-Wilk) and homogeneity (Levene’s test). Principal component analyses (PCA) of the FA profiles were performed for easier visualisation of complex, high-dimensional data, helping to identify patterns. To evaluate the significance of patterns observed (or their absence) in the PCA, differences in FA contents among treatments were analysed by one-way permutational multivariate analysis of variance (PERMANOVA), with “Diet” (Control, Inorganic Fe and Organic Fe) as the factor. Before conducting the analysis, data were square root transformed, the Euclidean similarity model was applied, and 9999 permutations were performed.

Differences in saturated fatty acids (SFA), monounsaturated fatty acids (MUFA), n-3 PUFA, n-6 PUFA, n-3 LC-PUFA, n-6 LC-PUFA and unsaturation index (UI; ∑ [area of fatty acid * number of unsaturations]), among treatments were tested by one-way ANOVA with Tukey’s post-hoc test for multiple comparisons. Statistical significance was assessed at 95% confidence level (*p* ≤ 0.05).

## Results

### Effects of iron on desaturase activity (*in vitro* assays)

**Fig. 2:**
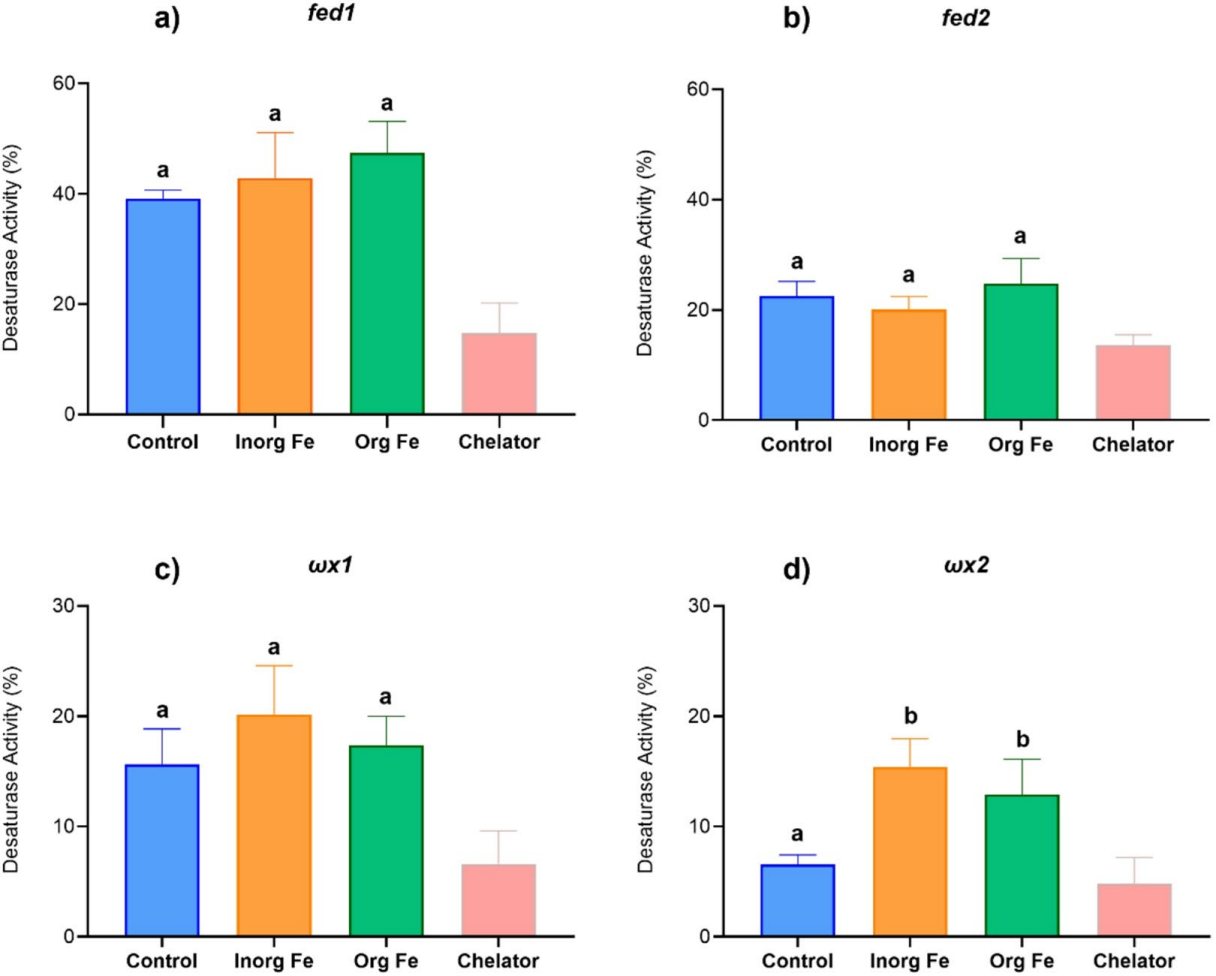
Effect of iron (Fe) supplementation on the activity of the front-end desaturases and methyl-end desaturases from *Hediste diversicolor* when expressed in yeast: **(a)** *fed1*, **(b)** *fed2*, **(c)** *ωx1* and **(d)** ωx2. Different treatments were tested: no Fe supplementation (Control, blue), supplementation with FeSO_4_ (Inorg Fe, orange), supplementation with ProPath^®^ Fe (Org Fe, green), and supplementation with an Fe chelating agent (Chelator treatment). Statistical differences (Dunnett’s multiple comparisons, *p* ≤ 0.05) are indicated by different lowercase letters. Each treatment was tested in four replicate 150 ml-flasks. Chelator treatment was excluded from statistical analysis (see text for more details).

The FA composition of yeast expressing the *H. diversicolor* desaturases and grown with supplementation of Fe as FeSO_4_ (Inorganic Fe) or ProPath^®^ Fe (Organic Fe) were compared to yeast cultured in the absence of exogenously added Fe. Additionally, a treatment consisting of yeast grown on a medium supplemented with an Fe chelating agent (Chelator) was used as negative control (Fig. 2). The FA analyses of the transgenic yeast clearly showed that all four enzymes were capable of desaturating the exogenously supplied substrate, although with varying conversions (Fig. 2). Moreover, our analyses revealed a reduction in desaturase activity in transgenic yeast expressing each of the four *H. diversicolor* desaturases, when grown in the presence of Fe chelator (Fig. 2). This treatment was therefore excluded from statistical analysis. The desaturase activities in yeast expressing the *H. diversicolor fed1*, *fed2* and *ωx1* and supplied with Fe (Inorganic Fe and Organic Fe) showed no significant differences (*p* > 0.05) compared to Control (Fig. 2a, b, c). Although no significant, a trend towards increased desaturase activity associated to Fe treatments was observed for yeast expressing *fed1* and *ωx1* (Fig. 2a, c). Interestingly, both Inorganic Fe and Organic Fe treatments resulted in significantly higher desaturase activity (*p* < 0.05) than Control for the *H. diversicolor ωx2* (Fig. 2d), with percentage conversion values of 6.56% in the Control, compared to 15.36% and 12.91% in the Inorganic Fe and Organic Fe treatments, respectively. This suggests that, under conditions promoting expression of the *H. diversicolor ωx2* as occurs in the herein used yeast system, this enzyme increases its desaturation capacity when Fe is exogenously supplied. Our results did not show any difference in desaturase activity of the *H. diversicolor ωx2* between the two Fe sources assayed.

### Growth and survival

Growth of polychaetes (SGR) was significantly affected by dietary treatment (one-way ANOVA, *p* ≤ 0.05). Specifically, statistically significant differences in SGR were observed between the Organic Fe (0.034) and Control (0.038) polychaetes (Table 2). The SGR reported for the Inorganic Fe polychaetes did not differ from the Control (Table 2). Survival averaged 96.1 ± 4.2% for all treatments and was not significantly affected by Fe treatments (one-way ANOVA, *p <* 0.05).

**Table 2:** Specific growth rates (SGR) calculated at the end of the trial (49 d). Values are expressed as the means ± SD (*n* = 58–60). Statistical differences were assessed using one-way ANOVA with tukey’s multiple comparisons (*p* ≤ 0.05) with different lowercase letters (a-b), indicating significant differences between treatments.

	Control	Inorganic Fe	Organic Fe
SGR	0.038 ± 0.000 ^a^	0.036 ± 0.001 ^ab^	0.034 ± 0.001 ^b^

### Effects of iron on desaturase activity *in vivo*

The efficiency of Fe supplementation on increasing desaturase activity was assessed by determining the FA profiles of *H. diversicolor* fed the experimental diets Control, Inorganic Fe and Organic Fe. The experimental diets were characterised by their richness in oleic acid (OA, 18:1n-9) (33.5–36.2%) and linoleic acid (LA, 18:2n-6) (14.4–16.7%), while lacking ARA (20:4n-6), EPA (20:5n-3) and DHA (22:6n-3) (Suppl. Table S2). Consequently, the experimental diets were rich in SFA and MUFA (35.0–45.0%), but lacked LC-PUFA (Suppl. Table S2).

Polychaetes at start of the experiment (Day 0) contained relatively high levels of LC-PUFAs, particularly EPA (10.2%) and DHA (10.9%) (Table 3), probably reflecting the impact of the marine ingredient-rich feed used prior the experiment. The FA composition of the worms changed remarkably over the experimental period, again as a result of the formulation of the experimental diets lacking LC-PUFAs (Table 3). Indeed, the FA composition of *H. diversicolor* from the three experimental treatments was characterised by elevated levels of typical plant-origin FAs such as OA and LA (Table 3). While the experimental diets lacked LC-PUFAs (Suppl. Table S2), the contents of LC-PUFAs in *H. diversicolor* fed those diets during 49 d included the n-3 LC-PUFAs EPA (3.1–3.7%) and DHA (0.7–0.8%), as well as the LC-PUFA n-6 ARA (2.5–2.8%) (Table 3).

**Table 3:** Fatty acid (FA) composition of samples of *H. diversicolor* at the beginning of the experiment (Day 0) and *H. diversicolor* fed the experimental diets (Control, inorganic Fe and organic Fe) for 49 d. Data are % of total FA (average ± SD, *n* = 6). NMI = non-methylene-interrupted fatty acids (e.g., 20:3 NMI).

FA	Day 0	Control	inorganic Fe	Organic Fe
14:0	2.8 ± 0.2	0.4 ± 0.3	0.3 ± 0.2	0.3 ± 0.2
16:0	17.6 ± 0.7	15.6 ± 1.9	16.5 ± 1.6	15.3 ± 1.6
16:1n-7	1.7 ± 0.2	0.7 ± 0.1	0.5 ± 0.3	0.6 ± 0.1
18:0	5.7 ± 0.4	3.9 ± 0.3	4.2 ± 0.7	3.9 ± 0.8
18:1n-13	3.6 ± 0.4	3.1 ± 0.6	2.5 ± 1.0	2.6 ± 1.0
18:1n-9 (OA)	8.2 ± 0.3	25.2 ± 2.1	26.1 ± 3.4	26.1 ± 3.3
18:1n-7	2.5 ± 0.1	1.3 ± 0.2	0.9 ± 0.2	0.8 ± 0.2
18:2n-6 (LA)	10.2 ± 0.5	21.8 ± 1.0	20.5 ± 1.3	21.0 ± 1.1
18:3n-3 (ALA)	1.2 ± 0.1	2.0 ± 0.4	2.1 ± 0.4	2.2 ± 0.4
20:1n-11	1.4 ± 0.2	1.1 ± 0.3	1.1 ± 0.3	1.2 ± 0.3
20:1n-9	2.7 ± 0.4	2.3 ± 0.3	2.2 ± 0.3	2.1 ± 0.4
20:3 nmi	1.5 ± 0.1	1.5 ± 0.3	0.9 ± 0.2	1.5 ± 0.3
20:2n-6	3.3 ± 0.3	4.6 ± 0.7	4.4 ± 0.9	4.4 ± 0.8
20:2 NMI	0.5 ± 0.1	1.1 ± 0.2	0.9 ± 0.2	1.1 ± 0.2
20:4n-6 (ARA)	1.3 ± 0.1	2.5 ± 0.3	2.5 ± 0.3	2.8 ± 1.0
20:5n-3 (EPA)	10.2 ± 0.4	3.1 ± 0.5	3.7 ± 1.0	3.4 ± 1.0
22:2 NMI	1.7 ± 0.2	1.3 ± 0.3	1.1 ± 0.3	1.2 ± 0.4
22:5n-3	0.9 ± 0.1	0.2 ± 0.1	0.3 ± 0.1	0.2 ± 0.1
22:6n-3 (DHA)	10.9 ± 0.7	0.8 ± 0.1	0.7 ± 0.2	0.7 ± 0.2
SFA	27.4 ± 0.9	20.4 ± 2.0	21.5 ± 1.8	20.0 ± 2.1
MUFA	23.5 ± 0.8	35.9 ± 1.1	35.2 ± 2.1	35.2 ± 2.4
n-3 PUFA	23.7 ± 0.4	6.5 ± 1.0	7.2 ± 1.3	6.8 ± 0.9
n-6 PUFA	16.2 ± 0.6	30.9 ± 1.4	29.0 ± 1.7	30.2 ± 1.7
n-3 LC-PUFA	22.6 ± 0.4	4.5 ± 0.9	5.1 ± 1.3	4.6 ± 1.1
n-6 LC-PUFA	6.1 ± 0.3	9.1 ± 0.8	8.6 ± 1.4	9.2 ± 1.8
UI	198.1 ± 2.3	140.8 ± 5.7	139.7 ± 8.1	140.3 ± 8.9

OA, oleic acid; LA, linolenic acid; ALA, alpha-linolenic acid; ARA, arachidonic acid; EPA, eicosapentaenoic acid; DHA, docosahexaenoic acid; NMI, non-methylene interrupted fatty acids; SFA, saturated fatty acids; MUFA, monounsaturated fatty acids; PUFA, polyunsaturated fatty acids; LC-PUFA, long-chain polyunsaturated fatty acids; UI, unsaturation index; ∑ [area of fatty acid * number of unsaturations]).

**Fig. 3:**
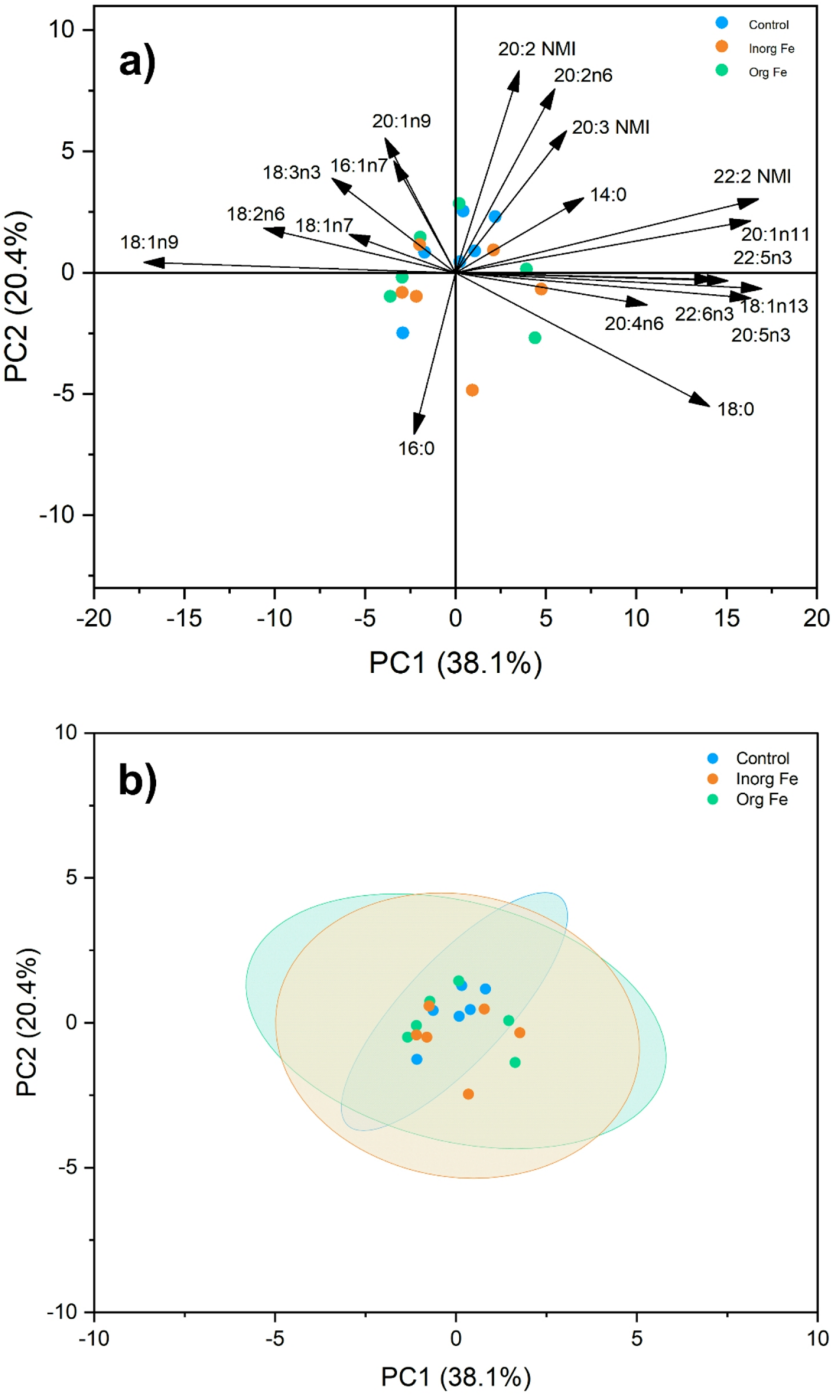
Principal Component Analysis (PCA) of the fatty acid composition of *Hediste diversicolor* (*n* = 6) fed with the experimental diets (Control, Inorganic Fe and Organic Fe**)** for 49 d. **(a)** loading plot and **(b)** score plot with 95% confidence ellipses.

The PCA of the FA composition of polychaetes showed no distinct clusters in the Fe supplementation treatments compared to the control (Fig. 3). The analysis indicated that the first component (PC1) accounted for 38.1% of the variance of this dataset, while PC2 accounted for 20.4% (Fig. 3). The PCA loading plot demonstrated a separation of LC-PUFAs, such as ARA (20:4n-6), EPA (20:5n-3) and DHA (22:6n-3), on the positive side of PC1, whereas OA (18:1n-9), LA (18:2n-6), and alpha-linolenic acid (ALA, 18:3n-3) loaded on the negative side of PC1 (Fig. 3a). The lack of differences on the FA profile of *H. diversicolor* among dietary treatments showed by PCA, was further examined using a one-way PERMANOVA. This multivariate analysis confirmed no significant differences in FA profiles between the polychaetes fed with Fe-supplemented diets and those of the control group (*F* = 0.627, *p* = 0.794).

### Gene expression *in vivo*

**Fig. 4:**
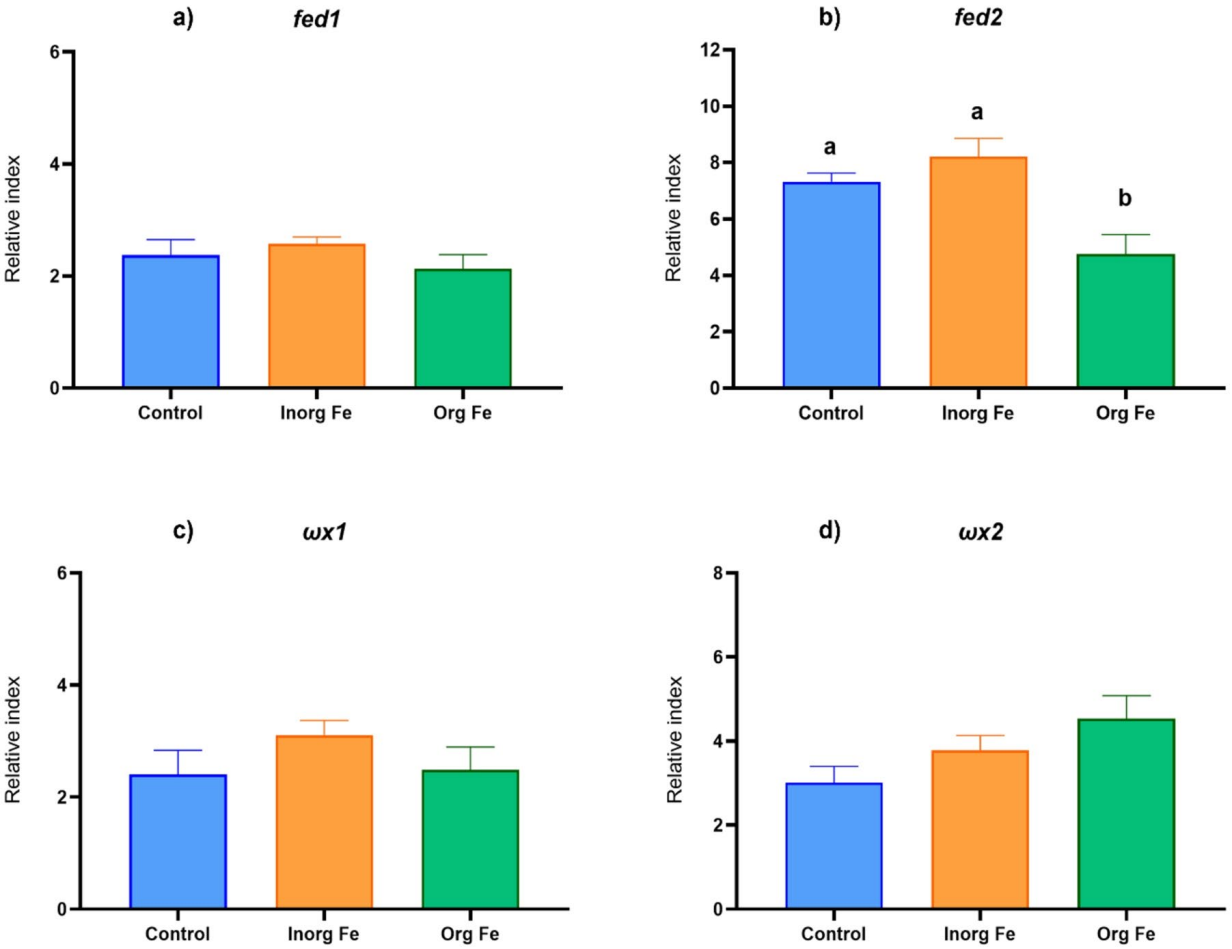
Relative gene expression of the front-end desaturases *fed1* **(a)** and *fed2* **(b)**, and the methyl-end desaturases *ωx1* **(c)** and *ωx2* **(d)** from *Hediste diversicolor* fed with the experimental diets (Control, Inorganic Fe and Organic Fe) for 49 d. Data are presented as mean ± SEM (*n* = 6). Statistical differences (Dunnett’s multiple comparisons, *p* ≤ 0.05) are indicated by different letters.

The gene expression of the front-end desaturases (*fed1* and *fed2*) and methyl-end desaturases from *H. diversicolor* fed the experimental diets was measured by qPCR (Fig. 4). The expression of *fed1* remained stable, showing no significant variation between the Fe treatments (Inorganic Fe and Organic Fe) and the Control (Fig. 4a). In contrast, *fed2* expression was similar between the Inorganic Fe and Control conditions, but exhibited a significant decrease (*p* < 0.05) under Organic Fe treatment compared to the Control (Fig. 4b). The expression levels of methyl-end desaturases (*ωx1* and *ωx2*) did not show significant differences between the Fe treatments (Inorganic Fe and Organic Fe) and Control (Fig. 4c and d).

## Discussion

Numerous studies have highlighted the importance of trace mineral supplementation in aquafeeds for both fish^38^ and crustaceans^27,39^. Fe plays a key role in LC-PUFA biosynthesis since it is a co-factor of fatty acyl desaturases involved in these pathways, and its dietary supplementation has suggested positive effects on LC-PUFA biosynthesis in salmonids^16–18^. Among invertebrates, Fe supplementation has been associated with improved growth performance in *E. sinensis* and *L. vannamei*^40,41^, but its potential to enhance LC-PUFA biosynthesis has remained unexplored.

The *in vitro* assays showed that Fe supplementation from both inorganic and organic Fe sources has potential to enhance the activity of fatty acyl desaturases involved in LC-PUFA biosynthesis under up-regulating conditions, as simulated in the yeast system. Notably, this effect was found significant for the *H. diversicolor* methyl-end desaturase ωx2, reported to be an ω3 desaturase enabling the bioconversion of a variety of n-6 substrates such as LA (18:2n-6) into n-3 products like ALA (18:3n-3)^8^. These findings are consistent with previous work in yeast, where Fe limitation reduced desaturase activity and ALA content^42^. Similar Fe-dependent responses have also been reported in microalgae and cyanobacteria, supporting the general role of Fe in regulating desaturation and lipid biosynthesis^43–46^. In the present study, although no significant differences were observed for yeast expressing *fed1* and *ωx1*, a trend towards higher activity under Fe supplementation suggests that Fe availability influences desaturase performance. Further studies could explore whether alternative Fe sources, including nanoparticle-based systems, produce similar effects.

The results from the *in vivo* feeding trial revealed no significant differences in the LC-PUFA composition of *H. diversicolor* across the experimental diets (Control, Inorganic Fe and Organic Fe), suggesting that the activity of the *H. diversicolor* fatty acyl desaturases did not differ among treatments. These findings in the polychaete *H. diversicolor* contrast with previous research on fish species like the rainbow trout (*O. mykiss*) fed on vegetal oil-based diets supplemented with Fe, found significantly higher levels of LC-PUFAs, particularly ARA, EPA and DHA, in the fish fillet^16^. While the specific contribution of Fe was not clarified, other studies on salmonids showed that fortification of micronutrients including Fe among others, resulted in a significant increase in DHA and total n-3 LC-PUFAs in whole-body fish^17,18^. Such an apparent discrepancy between our results and those from the fish studies discussed above suggests different regulatory mechanisms controlling LC-PUFA biosynthesis in annelids compared to fish. A robust body of evidence has shown that teleost fish in general, and salmonids in particular, have active compensatory mechanisms by which LC-PUFA biosynthesis is enhanced in response to a low dietary supply of LC-PUFAs linked to inclusion of plant ingredients in the feed^11^. The nutritional regulation of LC-PUFA biosynthesis in polychaetes has been barely explored, and our results suggest that the up-regulation of fatty acyl desaturases expected to occur by feeding *H. diversicolor* with a LC-PUFA-deficient diet did not take place. It is also important to note that, along potentially different regulatory mechanisms between fish and annelids, there exist obvious differences in the gene complement itself and, for instance, annelids possess methyl-end desaturases^47^, an enzyme type absent in vertebrates including fish. It is therefore tempting to hypothesise that the presence of methyl-end desaturases in polychaetes provides them with a more complete capacity for LC-PUFA biosynthesis compared to fish, potentially reducing their need to activate compensatory mechanisms when exposed to a low-LC-PUFA diets. As noted above, despite our feed formulation was designed to upregulate LC-PUFA biosynthesising genes, such activation might not have occurred to the extent we expected to. To confirm that the LC-PUFA-free diets tested in the present study elicited the expected up-regulation of fatty acyl desaturases, as suggested by our working hypothesis, a comparative analysis including a positive control group (e.g., worms fed a high LC-PUFA diet) would be required. The absence of such a reference group in the present experimental design represents a limitation, as it prevents a conclusive assessment of whether desaturase upregulation actually occurred *in vivo*. In the absence of this high-LC-PUFA dietary treatment in our experimental design, we cannot rule out the possibility that the free-LC-PUFA diets elicited an up-regulation the fatty acyl desaturase genes. Under this scenario, the Fe supplementation in both Inorganic Fe and Organic Fe did not result in increased LC-PUFAs compared to Control because Fe was not limiting to compromise the activity of the desaturase pool in any of the three treatments. It has been shown that *H. diversicolor* can meet its Fe requirements by direct absorption from the environment and/or ingestion of sediment, which naturally contains Fe among other trace minerals^48,49^. In addition to a particularly enhanced capacity for up taking Fe from the environment, it is also worth noting that some of the dietary components used the feed formulation, namely the plant protein sources and the micronutrient premix, might have provided enough Fe to guarantee the activity of the fatty acyl desaturases, even in the control treatment with no Fed supplementation.

The gene expression data collected in the present study suggests that Fe itself does not trigger the expression of fatty acyl desaturases involved in the LC-PUFA biosynthesis of *H. diversicolor*. These results are consistent with findings in Atlantic salmon, where the dietary over-fortification with cofactors including Fe, did not result in statistically significant differences in the expression of Δ6 and Δ5 desaturases^18^. This study demonstrated that, while some micronutrients including Fe are necessary for fatty acyl desaturation in Atlantic salmon, they do not appear to be a limiting factor, and dietary over-fortification did not improve, but rather hindered, fatty acyl desaturation^18^. In this context, the Fe supplementation levels used in our feeding trial (675 mg/kg diet) may have also been excessive, potentially compromising the activity of the fatty acyl desaturases in *H. diversicolor*, as observed in rats, where excess of Fe resulted in the downregulation of Δ5 and Δ6 desaturases^50–52^. In our experiment, *fed2* (Δ6/Δ8 desaturase) expression exhibited a significant decrease under Organic Fe treatment compared to the Control, suggesting that the higher bioavailability of chelated Fe may have led to excessive Fe accumulation and oxidative stress. This downregulation could reflect a physiological response to Fe overload, either through feedback inhibition or stress-related suppression of desaturase gene expression, both consistent with the reduced growth observed under Organic Fe supplementation. Similarly, research on *L. vannamei* revealed negative effects of high doses of inorganic Fe in their diet (955 mg/kg, provided as FeSO₄·7 H₂O) associated with increased hepatopancreas Fe deposition^40^. Such high dietary Fe concentrations are remarkably above the optimal Fe requirements, found to be, for example, between 130 and 141 mg/kg for the crab *E. sinensis*^41^. The Fe supplementation level in the present study was selected based on commercial recommendations for aquaculture feeds, given that no data on Fe dietary requirements are currently available for *H. diversicolor*. This represents the first attempt to evaluate Fe supplementation in this species. The reduced growth observed in worms fed the Organic Fe diet suggests that, due to the higher bioavailability of chelated Fe, excessive accumulation may have occurred, potentially reaching toxic levels that inhibited growth. Essential trace minerals such as Cu, Zn, and Fe are crucial for metabolic processes but can become toxic when exceeding tolerable limits^53–56^. Future studies should include measurements of Fe accumulation in worm tissues to clarify whether the observed effects were linked to Fe overload. While the exact reasons behind the reduced growth observed in our study with the Organic Fe treatment remain unclear, our results clearly indicate that dietary Fe concentrations must not exceed levels that can become toxic to *H. diversicolor*.

## Conclusions

The *in vitro* FA analysis indicated that Fe supplementation from both inorganic and organic sources enhanced the activity of desaturases involved in LC-PUFA biosynthesis under high expression conditions simulated in yeast. This effect was particularly noticeable on the *H. diversicolor* methyl-end desaturase ωx2. However, the *in vivo* analysis showed no significant differences in the LC-PUFA composition of *H. diversicolor* fed Fe-supplemented diets compared to controls. One possible explanation is that the LC-PUFA-free diet did not sufficiently up-regulated the *H. diversicolor* fatty acyl desaturases, meaning there was no increased Fe demand to ensure the activity of the enzymes. Alternatively, while the LC-PUFA-free diet may have up-regulated desaturases as hypothesised, the effects of Fe supplementation and the expected increase in desaturase activity were not apparent in the LC-PUFA contents of *H. diversicolor* fed either the Inorganic Fe or Organic Fe diets. This suggests that Fe was not a limiting factor even in Control. The findings highlight the importance of carefully monitoring dietary Fe concentrations to, on one hand, meet the requirements and, on the other, prevent potential detrimental effects to *H. diversicolor*, particularly when highly bioavailable sources are used.

## Supplementary Information

Below is the link to the electronic supplementary material.

Supplementary Material 1

## Data Availability

Data supporting this study will be made available upon reasonable request. Please contact the corresponding author at oscar.monroig@csic.es.
