# Supplementary material for: Investigating the potential of dietary iron supplementation to enhance long-chain polyunsaturated fatty acid biosynthesis in Hediste diversicolor
Source: Sci Rep. 2025 Nov 27;15:42473. doi: 10.1038/s41598-025-26488-x (PMC12660862; doi:10.1038/s41598-025-26488-x)
Supplement: Supplementary file 1 — Supplementary Material 1 [file 41598_2025_26488_MOESM1_ESM.docx]

**Supporting Information**

**Supplementary Table S1**. Ingredient composition of the experimental diets as dry matter (DM) basis (%). Note that diets for the Inorganic Fe and Organic Fe treatments included 675 mg kg^-1^ or FeSO_4_ and ProPath® Fe, respectively.

| Ingredients | % DM |
| --- | --- |
| CPSP90* | 1.0 |
| Wheat gluten (80% protein) | 19.0 |
| Soycomil (65% protein) | 28.0 |
| Pea concentrate (90% protein) | 10.0 |
| palm oil | 8.1 |
| Maltodextrin | 30.7 |
| Betaine | 0.5 |
| Vitamin and mineral premix | 0.5 |
| Vitamin C | 0.1 |
| guar Gum | 2.0 |

*Fish hydrolysate used as attractant.

Fe supplementation levels were 0 mg Fe kg⁻^1^ (Control), 135 mg Fe kg⁻^1^ (Inorganic Fe), and 101 mg Fe kg⁻^1^ (Organic Fe).

**Supplementary Table S2**. Fatty acid (FA) composition (% of total FA) of the experimental diets (Control, Inorganic Fe and Organic Fe) used in the trial. Data are % of total fatty acids (average ± SD, n = 4).

| FA | Control | inorganic Fe | Organic Fe |
| --- | --- | --- | --- |
| 14:0 | 0.9 ± 0.1 | 0.8 ± 0.0 | 0.8 ± 0.0 |
| 15:0 | 0.1 ± 0.0 | 0.1 ± 0.0 | 0.1 ± 0.0 |
| 16:0 | 38.0 ± 1.1 | 37.9 ± 0.8 | 38.8 ± 0.7 |
| 16:1n-7 | 0.3 ± 0.0 | 0.2 ± 0.0 | 0.2 ± 0.0 |
| 17:0 | 0.1 ± 0.0 | 0.1 ± 0.0 | 0.1 ± 0.0 |
| 18:0 | 4.5 ± 0.2 | 4.6 ± 0.1 | 4.7 ± 0.1 |
| 18:1n-9 (OA) | 33.5 ± 0.2 | 35.3 ± 0.3 | 36.2 ± 1.2 |
| 18:2n-6 (LA) | 16.7 ± 1.0 | 16.1 ± 0.8 | 14.4 ± 1.4 |
| 18:3n-3 (ALA) | 0.9 ± 0.1 | 0.8 ± 0.1 | 0.3 ± 0.3 |
| 20:0 | 0.4 ± 0.0 | 0.4 ± 0.0 | 0.4 ± 0.0 |
| 20:1n-9 | 0.3 ± 0.0 | 0.2 ± 0.1 | 0.3 ± 0.0 |
| SFA | 44.2 ± 1.4 | 44.0 ± 0.8 | 45.0 ± 0.8 |
| MUFA | 35.0 ± 0.6 | 36.6 ± 0.2 | 37.8 ± 0.9 |
| n-3 PUFA | 0.9 ± 0.1 | 0.8 ± 0.1 | 0.4 ± 0.3 |
| n-6 PUFA | 16.8 ± 1.0 | 16.1 ± 0.7 | 14.4 ± 1.4 |
| n-3 LC-PUFA | n.d. | n.d. | n.d |
| n-6 LC-PUFA | n.d. | n.d. | n.d. |
| UI | 71.8 ± 1.5 | 71.8 ± 1.2 | 68.0 ± 2.7 |

OA, oleic acid; LA, linolenic acid; ALA, a-linolenic acid; ARA, arachidonic acid; EPA, eicosapentaenoic acid; DHA, docosahexaenoic acid; SFAs, saturated fatty acids; MUFA, monounsaturated fatty acids; PUFA, polyunsaturated fatty acids; LC-PUFA, long-chain polyunsaturated fatty acids; UI, unsaturation index. n.d., not detected
